# Metabolic Changes in Serum and Milk of Holstein Cows in Their First to Fourth Parity Revealed by Biochemical Analysis and Untargeted Metabolomics

**DOI:** 10.3390/ani14030407

**Published:** 2024-01-26

**Authors:** Zixin Liu, Aoyu Jiang, Xiaokang Lv, Chuanshe Zhou, Zhiliang Tan

**Affiliations:** 1 CAS Key Laboratory for Agri-Ecological Processes in Subtropical Region, National Engineering Laboratory for Pollution CON and Waste Utilization in Livestock and Poultry Production, Hunan Provincial Key Laboratory of Animal Nutrition Physiology and Metabolic Process, Institute of Subtropical Agriculture, Chinese Academy of Sciences, Changsha 410125, China; liuzixin20@mails.ucas.ac.cn (Z.L.); jiangaoyu21@mails.ucas.ac.cn (A.J.); 13121191399@163.com (X.L.); zltan@isa.ac.cn (Z.T.); 2 University of Chinese Academy of Sciences, Beijing 100049, China; 3 College of Animal Science, Anhui Science and Technology University, Bengbu 233100, China

**Keywords:** lactation, dairy cattle, lactose, amino acids, metabolites, precision livestock farming

## Abstract

**Simple Summary:**

The body metabolic status of dairy cows is closely related to lactation performance. Their production performance will change with their years of service, and the milk quality also varies among different parities. However, there is still lack of research on the relationship between the metabolic state of Holstein cows and the performance of lactation across multiple parities. This study’s findings uncover the connection between the serum metabolome of Holstein cows and milk production. The results indicate variations in the lactation energy among the Holstein cows at different parities. Blood metabolites emerge as pivotal regulators of the milk yield and quality in the Holstein cows, underscoring the significance of addressing nutritional needs during these stages to enhance lactation performance. It is essential to understand the metabolic changes in Holstein cows at different parities to develop more refined feeding strategies, improve management methods, and strengthen the health of the cows.

**Abstract:**

The performance of dairy cows is closely tied to the metabolic state, and this performance varies depending on the number of times the cows have given birth. However, there is still a lack of research on the relationship between the metabolic state of Holstein cows and the performance of lactation across multiple parities. In this study, biochemical analyses and metabolomics studies were performed on the serum and milk from Holstein cows of parities 1–4 (H1, N = 10; H2, N = 7; H3, N = 9; H4, N = 9) in mid-lactation (DIM of 141 ± 4 days) to investigate the link between performance and metabolic changes. The results of the milk quality analysis showed that the lactose levels were highest in H1 (*p* = 0.036). The total protein content in the serum increased with increasing parity (*p* = 0.013). Additionally, the lipase activity was found to be lowest in H1 (*p* = 0.022). There was no difference in the composition of the hydrolyzed amino acids in the milk among H1 to H4. However, the free amino acids histidine and glutamate in the serum were lowest in H1 and highest in H3 (*p* < 0.001), while glycine was higher in H4 (*p* = 0.031). The metabolomics analysis revealed that 53 and 118 differential metabolites were identified in the milk and serum, respectively. The differential metabolites in the cows’ milk were classified into seven categories based on KEGG. Most of the differential metabolites in the cows’ milk were found to be more abundant in H1, and these metabolites were enriched in two impact pathways. The differential metabolites in the serum could be classified into nine categories and enriched in six metabolic pathways. A total of six shared metabolites were identified in the serum and milk, among which cholesterol and citric acid were closely related to amino acid metabolism in the serum. These findings indicate a significant influence of blood metabolites on the energy and amino acid metabolism during the milk production process in the Holstein cows across 1–4 lactations, and that an in-depth understanding of the metabolic changes that occur in Holstein cows during different lactations is essential for precision farming, and that it is worthwhile to further investigate these key metabolites that have an impact through controlled experiments.

## 1. Introduction

Holstein cows, widely distributed across the globe, are the preeminent breed of dairy cattle, renowned for their prodigious milk production capacity, which far surpasses that of other dairy breeds. Moreover, the Holstein cow exhibits an exceptional ability to maintain its milk production levels over many lactation cycles, which have been shown to improve milk quality with each successive lactation [1]. Several studies have reported that milk production is generally higher in multiparous cows than in primiparous cows [2,3,4], along with having a higher milk protein and fat content [3]. Furthermore, parity has also been found to impact the composition of milk fatty acids in Holstein cows. Milk from primiparous cows has a relatively high proportion of unsaturated fatty acids that are beneficial to humans. In comparison to multiparous cows, milk fat contains a lower proportion of fatty acids associated with high cholesterol, as well as saturated fatty acids, which may be less favorable to human health [5,6]. Despite this remarkable physiological trait, lactation in Holstein cows is a complex biological process governed by a multitude of intricate metabolic alterations [7]. A comprehensive understanding of the metabolic changes occurring in the serum and milk of Holstein cows without any experimental treatment during distinct lactation periods is currently lacking. In addition, ensuring that high milk production levels and quality are sustained throughout successive lactation cycles poses a significant challenge, as the metabolic demands required for optimal milk production exhibit temporal variability.

In the past few years, there have been significant advancements in metabolomics technology, which has enabled a more thorough and efficient analysis of metabolites in biological samples [8]. These metabolites are the end products of various metabolic pathways and can provide crucial information about the metabolic state of an organism. By measuring the levels of multiple metabolites simultaneously, metabolomics can provide a more comprehensive and detailed picture of the metabolic changes occurring in an animal organism [9], which is potentially beneficial in the field of dairy farming for controlling the quality of meat and milk [10]. One of the significant advantages of metabolomics is its ability to provide a global snapshot of the metabolic state of an organism [11]. As a result, it has emerged as a powerful tool for studying the metabolic profile of biological systems.

The metabolic changes in dairy cows during lactation were investigated in prior research efforts, with a focus on changes in the body fluids of dairy cows such as serum, rumen fluid, urine, or milk [10,12,13,14,15]. Studies have employed advanced metabolomics techniques such as nuclear magnetic resonance spectroscopy, mass spectrometry, and high-performance liquid chromatography to identify numerous metabolites that are significantly altered during lactation, including amino acids, carbohydrates, lipids, and organic acids [16,17]. These metabolites play critical roles in the metabolic adaptations that occur during lactation, such as energy metabolism, lipid metabolism, and protein metabolism [13]. For example, compared with primiparous cows, the serum metabolome showed that multiparous cows had more active fat mobilization with phosphatidylcholine carrying fatty acid moieties ranging from 28 to 36 carbons were enhanced in cows displaying high lipid mobilization, whereas the quantity of PC containing larger fatty acid components (≥40 carbons) was reduced [18]. Primiparous cows had an overall 40% increase in the serum levels of acylcarnitines and some phosphatidylcholines compared to multiparous cows [19]. Nevertheless, the scope of these investigations has often been constrained, emphasizing particular metabolites or select temporal phases. Therefore, there is a necessity to conduct more comprehensive and wide-ranging studies that investigate the metabolic changes that manifest in Holstein cows across multiple parities, aiming to identify the critical metabolites that underlie these transformations.

In this study, we aimed to investigate the metabolic changes in the serum and milk of Holstein cows during different parities using metabolomics and a conventional nutritional approach. Specifically, we aimed to identify the key metabolites involved in the metabolic changes occurring during different parities and to gain insights into the adaptations of the lactation metabolic status over time. By understanding the metabolic changes occurring during different parities, we hope to provide valuable information for optimizing management practices and improving milk production and cow health.

## 2. Materials and Methods

### 2.1. Animals and Experimental Design

The experimental procedures were approved by the Animal Care Committee at the Institute of Subtropical Agriculture (Changsha, China) and were in accordance with the Chinese Academy of Sciences’ guidelines for animal research. The experimental Holstein cows in this study were sourced from Hulunbuir Agricultural Reclamation Xiertala Farm (49°18′58.83″ N, 119°56′13.67″ E) and Ranch Co., Ltd., located in Hulunbuir City, Inner Mongolia Autonomous Region, China. Thirty-five healthy Holstein cows in their first to fourth parity with similar lactation stages were enrolled and allocated into four groups. The groups were labelled H1, H2, H3, and H4, with H1 consisting of 10 first-parity Holstein cows with an average days in milk (DIM) of 143 ± 3 (Mean + SD) days, H2 comprising 7 s-parity Holstein cows with an average DIM of 139 ± 5 days, H3 comprising 9 third-parity Holstein cows with an average DIM of 144 ± 3 days, and H4 comprising 9 fourth-parity Holstein cows with an average DIM of 136 ± 4 days. These cows were fed with total mixed rations (TMR) three times per day and had ad libitum access to water. The basal diet ingredients and nutrient composition are provided in Table S1. Milk yield data were collected using the Cascade™ parallel milking system (DeLaval, Sweden). Milk was collected from each cow three times per day, at 0530, 1330, and 1930 h.

### 2.2. Sample Collection and Analysis

Feed intake and refusal weights were measured daily, and total mixed ration (TMR) and refusal samples were collected during a five-day sampling period for nutrient level analysis. Feed samples were dried in an oven at 65 °C for 72 h and subsequently ground through a 1 mm sieve using a Wiley Mill (Thomas Scientific). The gross energy (GE) was determined using an isothermal automatic calorimeter 5E-AC8018 (Kaiyuan Instruments Co., Ltd., Changsha, China). The dry matter (DM), crude protein (CP), ether extract (EE), ash, calcium (Ca), and phosphorus (P) were analyzed using methods outlined by the Association of Official Analytical Chemists (AOAC, 2002). Neutral detergent fiber (NDF) and acid detergent fiber (ADF) were measured using an automatic fiber analyzer FT12 (Gerhardt, Bonn, Germany), according to Van Soest et al. [20]. 

Blood samples were obtained from the tail vein using a 20-gauge × 2.54 mm needle one hour before morning feeding. The samples were collected in 5 mL vacutainer tubes and immediately placed on ice until centrifuged at 3000 rpm at 4 °C for 15 min. The upper 2 mL of serum was collected from each tube and divided into three portions, and immediately frozen in liquid nitrogen and stored at −80 °C for subsequent analysis of serum biochemical parameters, free amino acid determination, and metabolomics analysis. In this study, H1-B, H2-B, H3-B, and H4-B represented the group names of serum samples from the H1–H4 groups, respectively. Serum biochemical parameters, including glucose (GLU), albumin (ALB), total protein (TP), blood urea nitrogen (BUN), triglyceride (TG), high-density lipoprotein cholesterol (HDL-C), low-density lipoprotein cholesterol (LDL-C), lipase (LIP), and blood ammonia (NH_3_L), were analyzed using commercial kits (Meikang Biotechnology Co., Ltd., Ningbo, China) with an automatic biochemical analyzer (Hitachi 7600, Tokyo, Japan). Serum free amino acids were determined using post-column derivatization. After mixing 1 mL of 8% sodium sulfosalicylate was added to 1 mL of serum sample to precipitate the serum protein and centrifugation at 10,000 rpm for 5 min, 1 mL of the supernatant was passed through a 0.22 μm filter membrane and analyzed using an amino acid analyzer (Hitachi L8900, Tokyo, Japan).

The milk samples were grouped as H1-M, H2-M, H3-M, and H4-M, corresponding to the S1–S4 groups of the Holstein cows. Milk and blood samples were collected on the same day. At three time points—0530, 1330, and 1930 h, 50 mL of milk was collected and mixed in the ratio of 4:3:3. The mixed milk was separated into three portions: one portion was added with potassium dichromate and stored at 4 °C for milk quality assessment, and the other two portions were immediately frozen in liquid nitrogen and stored at −80 °C for subsequent hydrolyzed amino acid determination and metabolomic analysis, respectively. The quality of milk samples was assessed using Basic Unit MilkoScan FT +200 Type 76150 (Foss Electric, Hillerod, Denmark). To determine the hydrolyzed amino acids, 6 mol/L hydrochloric acid (HCL) was mixed with the milk samples in a 1:1 (*v*:*v*) ratio in an ampoule bottle, which were then hydrolyzed at 110 °C for 24 h. The hydrolyzed solution was diluted to 50 mL, and 1 mL was taken to dry and remove the solvent. The remaining residue was dissolved in 1 mL of 0.01N HCl, filtered through a 0.22 μm filter membrane, and the hydrolyzed amino acids were quantified using an amino acid analyzer (Hitachi L8900, Tokyo, Japan).

### 2.3. Metabolomics Analysis by UPLC-MS/MS

Samples of serum and milk were prepared for LC-MS analysis by initially thawing them at 4 °C and adjusting any insufficient samples to an equal scale. Next, 100 µL of each sample was transferred into 2 mL centrifuge tubes, followed by the addition of 100 µL of mixed internal standard solution and 400 µL of −20 °C methanol. The samples were vortexed for 60 s and then centrifuged at 12,000 rpm and 4°C for 10 min. The supernatant from each sample was transferred into another 2 mL centrifuge tube, and the samples were concentrated to dryness in vacuum. The samples were then dissolved in 150 μL of 80% methanol solution and centrifuged at 12,000 rpm and 4 °C for 10 min to obtain the supernatant for LC-MS analysis. Quality control (QC) samples were prepared by taking 20 µL from each sample and were used to monitor deviations from the analytical results of the pool mixtures and the analytical instrument itself. The remaining samples were used for LC-MS detection. Chromatographic separation was performed using an ACQUITY UPLC^®^ HSS T3 column (150 × 2.1 mm, 1.8 µm, Waters) held at 40 °C, with the autosampler temperature maintained at 8 °C. Gradient elution of analytes was achieved with either 0.1% formic acid in water (C) and 0.1% formic acid in acetonitrile (D), or 5 mM ammonium formate in water (A) and acetonitrile (B), at a flow rate of 0.25 mL/min. Equilibrated samples were injected with a volume of 2 μL. The linear gradient of solvent B (*v*/*v*) was incrementally increased. ESI-MSn experiments were conducted in positive and negative modes, with a spray voltage of 3.5 kV and −2.5 kV, respectively. Sheath and auxiliary gases were set to 30 and 10 arbitrary units, and the capillary temperature was maintained at 325 °C. The Orbitrap analyzer scanned a mass range of m/z 100–1000 for full scans at a resolution of 60,000. HCD scan DDA MS/MS experiments were carried out with cracking rates of 30, 50, and 150%, while dynamic exclusion was implemented to remove unnecessary information in MS/MS spectra.

### 2.4. Metabolomics Data Processing

The acquired raw data were converted into mzXML format (xcms input file format) using Proteowizard software (v3.0.8789). Peak identification, filtration, and alignment were performed using the XCMS program package in R (v3.3.2), with primary parameters including bw = 5, ppm = 15, peakwidth = c (5, 30), mzwid = 0.015, mzdiff = 0.01, and method = “centWave”. This resulted in a data matrix containing information such as mass-to-charge ratio (*m*/*z*), retention time, and peak intensity. A total of 14,397 precursor molecules were obtained in positive ion mode, and 5723 in negative ion mode, which were exported to Excel for subsequent analysis. To facilitate comparison of data across different scales, batch normalization of peak areas was performed. The resulting data matrices underwent mean-centering and were scaled to unit variance (UV). Then, SIMCA-P software (Version 15.0, Umetrics AB, Umeå, Sweden) and the R package RoPLS were employed to conduct orthogonal partial least-square discriminant analysis (OPLS-DA). The validity of the models was determined by total explained variance (R2) and predictability (Q2). Differential metabolites were screened based on molecular weight error < 15 ppm, Variable Importance for the Projection (VIP) > 1, and one-way ANOVA calculated *p*-value ≤ 0.05. Accurate information of metabolites was obtained by further matching and annotating them in the Metlin (http://metlin.scripps.edu (accessed on 21 July 2021)), MoNA (https://mona.fiehnlab.ucdavis.edu/ (accessed on 21 July 2021)), and the database built by BioNovoGene Co., Ltd. (Suzhou, China). Hierarchical clustering analysis was performed using the agglomerate hierarchical clustering method, and the dataset was scaled and normalized using the pheatmap package in R (v3.3.2). Furthermore, differential metabolites were identified and categorized using the Kyoto Encyclopedia of Genes and Genomes (KEGG) pathway database (http://www.genome.jp/kegg/ (accessed on 25 May 2022)). Metaboanalyst 5.0 (http://www.metaboanalyst.ca/ (accessed on 25 May 2022)) was employed for metabolic impact pathway analysis. Venn diagrams were performed using the OmicStudio tools at https://www.omicstudio.cn (accessed on 4 November 2023). Associations between shared metabolites in serum and milk were analyzed by Spearman’s correlation using the vegan package in R (vesion 4.3.1). Correlation networks consisting of differential metabolites with differential indicators detected in serum and milk were tested analytically using Mantel’s test, Pearson’s correlation, and visualized using the R (vesion 4.3.1) packages linkET and ggplot2. 

### 2.5. Statistical Analysis

The preliminary data processing was conducted using Excel 2019 (Microsoft Corporation, Redmond, WA, USA). The milk quality, serum biochemical parameters, serum free amino acids, and milk hydrolyzed amino acids were assessed for normality using the Shapiro–Wilk test, and then compared using ANOVA with Bonferroni adjustment for multiple comparisons between categorical variables. Statistical analysis was performed using Statistical Package for the Social Sciences 22.0 software (SPSS, Inc., Armonk, NY, USA), with significance set at *p* ≤ 0.05 and trends observed at 0.05 < *p* < 0.10.

## 3. Results

### 3.1. Milk Quality and Hydrolyzed Amino Acids

The production performance results of H1–H4 are shown in Table 1. In the case of no difference in the BW among the different parities (*p* > 0.05), the DMI showed an increasing trend with the parity (linear, *p* = 0.052), and the milk yield did not change significantly with the parity (*p* > 0.05). For the milk quality, there was a significant quadratic variation in the lactose among the different parity cows (quadratic, *p* = 0.009); the H1 content was the highest, and the 2/3 parity was significantly lower than in H1 (*p* = 0.036). The variation trend of the urea nitrogen among the parities was opposite to that of lactose, which was significantly higher in the 2/3 parities than H1 and H4 (quadratic, *p* = 0.021). However, other indicators related to milk quality, such as the somatic cell count, milk fat, milk protein, and dry matter without fat did not change significantly between H1 and H4 (*p* > 0.05). The protein composition in the milk was further analyzed, and the results of the milk hydrolyzed amino acids between the different parities of the Holstein cows are shown in Table S2. Neither the nine EAAs, including arginine, threonine, valine, methionine, isoleucine, leucine, phenylalanine, lysine, and histidine, nor the seven NEAAs, including aspartate, serine, glutamate, glycine, alanine, tyrosine, and proline, showed any significant differences (*p* > 0.05).

### 3.2. Serum Biochemical Parameters and Free Amino Acids

The results of the serum biochemical parameters of the 1–4 parities in the Holstein dairy cattle are shown in Table 2. The glucose content showed an increasing trend with increasing parity (*p* = 0.081); the total protein also showed a significant linear variation (linear, *p* = 0.002) and was highest in H4 (*p* = 0.013), while the activity of lipase was significantly lower in H1 than in the other three parities of the Holstein cows (*p* = 0.022). In addition, there were no significant differences in albumin, blood urea nitrogen, triglyceride, low-density lipoprotein, high-density lipoprotein, and serum ammonia among the Holstein cows of 1–4 parity (*p* > 0.05). The free amino acid content in the serum was further determined and the results are shown in Table 3. Among them, histinine, belonging to the EAAs, and glutamate and glycine, belonging to the NEAAs, showed significant differences in the serum of the 1–4 parities in the Holstein cows. Histinine (*p* < 0.001) and glutamate (*p* = 0.039) were both lowest in H1 and highest in H3, while glycine (*p* = 0.031) was highest in H4. Serine in the H1–H4 serum showed a linear increase with the parity (*p* = 0.011), and EAA/TAA was observed in a quadratic increase (*p* = 0.027), which was higher in H2 and H3. Except for the amino acids mentioned above, there were no significant differences in the other serum free amino acids in H1–H4.

### 3.3. Metabolomics Multivariate Analysis of Serum and Milk

**Figure 1:**
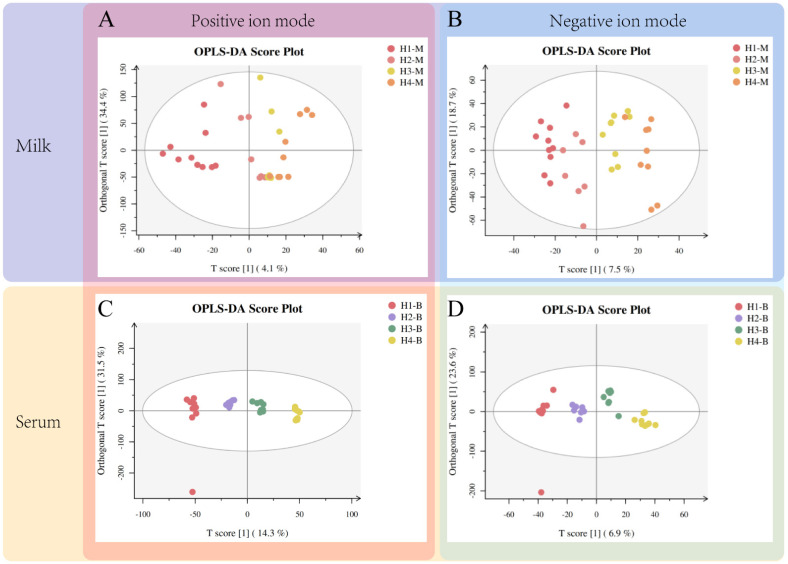
Orthogonal partial least square discriminant analysis (OPLS-DA) score plot for the serum and milk samples from Holstein dairy cattle of different parities. The vertical pink and blue blocks represent the positive and negative modes, respectively. The horizontal wheat and purple blocks represent serum and milk samples, respectively. Triangles of different colors represent samples from corresponding groups. T score [1] represents the regression coefficient weight of the abscissa, and Orthogonal T score [1] represents the regression coefficient weight of the ordinate.

The analysis of the UPLC-MS/MS off-line data revealed that, in the cationic mode, the score plots from the OPLS-DA showed that some individual milk samples (T score [1] = 4.1%, Orthogonal T score [1] = 34.4%) were closer together (Figure 1A), but in the anionic mode (T score [1] = 7.5%, Orthogonal T score [1] = 18.7%) could be significantly separated (Figure 1B). The OPLS-DA score plots of the serum samples showed that H1–H4 were well homogenized and tightly aggregated within the group and differed significantly between groups, and both in the positive mode (Figure 1C, T score [1] = 14.3%, Orthogonal T score [1] = 31.5%) and in the negative mode (Figure 1D, T score [1] = 6.9%. Orthogonal T score [1] = 23.6%), all four groups were completely separated. The multivariate statistical analysis parameters for the OPLS-DA models from the serum and milk are listed in Table S3. 

### 3.4. Differential Metabolite Identification of Serum and Milk

**Figure 2:**
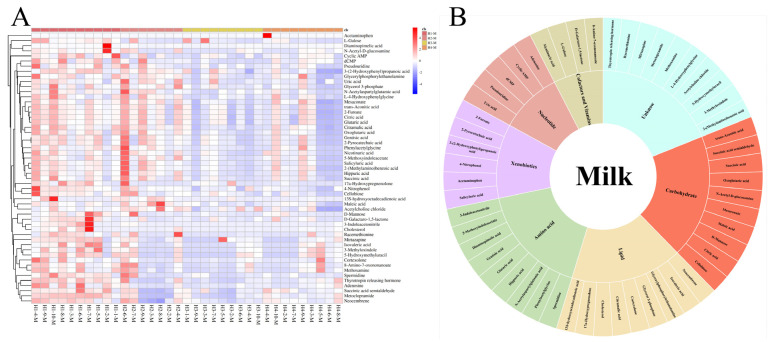
(**A**) Hierarchical cluster analysis (HCA) of differential metabolites identified from H1-M, H2-M, H3-M, and H4-M. (**B**) Classification of metabolites from milk based on KEGG.

**Figure 3:**
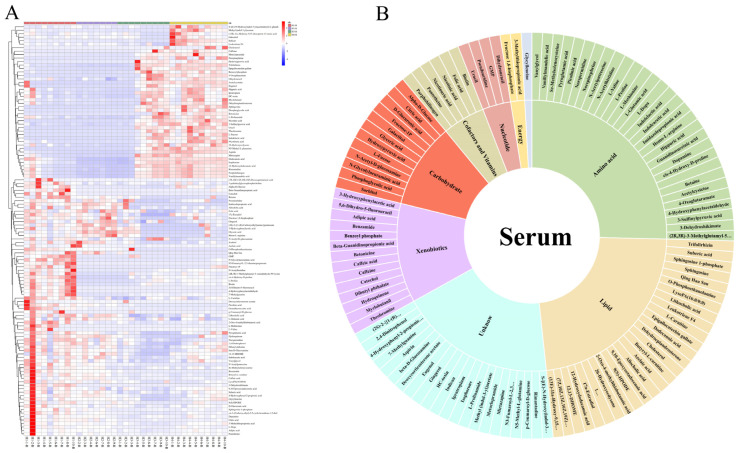
(**A**) Hierarchical cluster analysis (HCA) of differential metabolites identified from H1-B, H2-B, H3-B, and H4-B. (**B**) Classification of metabolites from serum based on KEGG.

The VIP of the OPLS-DA models were applied to filter the significant metabolites; a VIP value > 1 and *p*-value < 0.05 were considered statistically significant. A total of 53 differential metabolites were screened in the milk of the Holstein cows at parities 1–4, and the specific names, mass-to-charge ratio, retention time, molecular formula, and screening mode of these metabolites are listed in Table S4. The same criteria screened 118 differential metabolites in the serum from H1–H4, and information on these metabolites is presented in Table S5. To further explore the distribution of these differential metabolites across different parities, a hierarchical cluster analysis (HCA) was performed for the differential metabolites identified in the milk and serum. The heat map results showed that the identification intensity of these differential metabolites were higher in the H1 milk, and some of them also had a higher identification intensity in H2, while H3 and H4 were generally low (Figure 2A). The classification of these differential metabolites based on the KEGG database revealed a total of seven superpathways of metabolism that could be classified into amino acid metabolism, lipid metabolism, xenobiotic metabolism, nucleotide metabolism, carbohydrate metabolism, cofactor and vitamin metabolism, and the unknown class (Figure 2B). The heat map of the differential metabolites from the serum showed that the metabolic patterns of H3 and H4 were more similar, while H1 and H2 were different to the other groups (Figure 3A). The same classification of these differential metabolites suggests that in addition to the seven classes that can be assigned to milk, the differential metabolites from the serum can be assigned to two additional superpathways, energy metabolism and peptide metabolism, for a total of nine categories (Figure 3B).

### 3.5. Pathway Analysis of Serum and Milk

**Figure 4:**
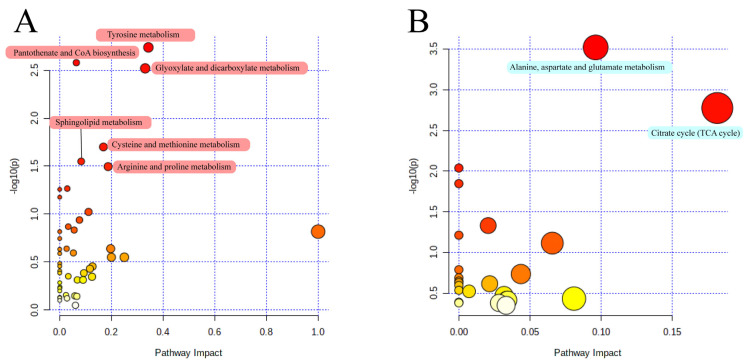
(**A**) Functional pathway enrichment analysis of differential metabolites from serum. (**B**) Functional pathway enrichment analysis of differential metabolites from milk. The colour and size of the bubbles indicate the *p*-value and the pathway impact index, the darker the bubble the higher the *p*-value and the larger the bubble the higher the pathway impact index.

Based on the KEGG database, after the enrichment and pathway topology analysis of the differential metabolites identified in the milk and serum, two and six impact pathways were identified, respectively, combined with the impact value at the comprehensive level (Figure 4). In the milk (Figure 4A), we observed metabolic pathways related to alanine, aspartate, and glutamate metabolism, as well as the citrate cycle (TCA cycle). In the serum (Figure 4B), the identified pathways included tyrosine metabolism, pantothenate and CoA biosynthesis, glyoxylate and dicarboxylate metabolism, cysteine and methionine metabolism, sphingolipid metabolism, and arginine and proline metabolism.

### 3.6. Interaction Analysis of Serum and Milk Metabolites

**Figure 5:**
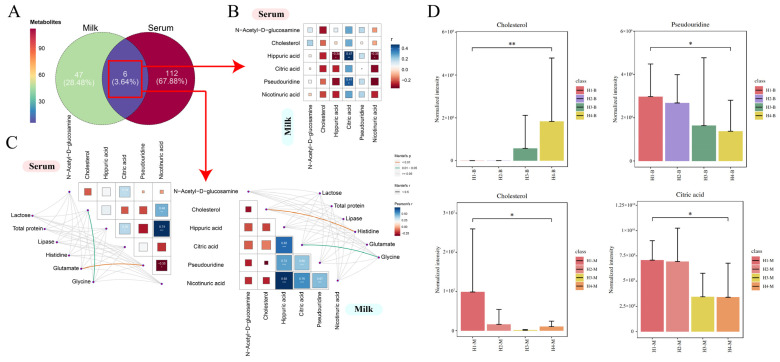
(**A**) Venn diagram of the distribution of shared differential metabolites of serum and milk for H1–H4. Green circles indicate milk, red circles indicate serum, and purple circles indicate milk shared with serum. (**B**) Spearman’s correlation analysis of shared differential metabolites in serum and milk from H1–H4. Horizontal coordinates indicate shared metabolites in milk, vertical coordinates indicate shared metabolites in serum, * represents 0.01 < *p* < 0.05, ** represents 0.001 < *p* < 0.01, and *** represents *p* < 0.001. (**C**) Correlation network analysis of shared differential metabolites with indicators of difference in serum and milk. On the left, the associations of 6 shared differential metabolites with significant indicators of difference in H1–H4 serum are shown. On the right, the associations of 6 shared differential metabolites with significant indicators of difference in H1–H4 milk are shown. Correlations between metabolites and differential indicators were determined using Mantel’s tests, with the thickness of the connecting line indicating the correlation coefficient, with a thick line indicating a Mantel’s r ≥ 0.5; the color of the connecting line indicated significance, with orange being a highly significant correlation (*p* < 0.01) and green being a significant correlation (0.01 < *p* < 0.05). Pearson’s test tested correlations between shared differential metabolites; box size and color gradient indicate Pearson’s correlation, blue indicates positive correlation, red indicates negative correlation, and white words in the boxes indicate correlation coefficients. (**D**) Intensity of identification in serum or milk from H1–H4 of important metabolites significantly associated with differential physiological indicators.

To further explore the correlation between the differential metabolites in the cow serum and milk, it was found that six (3.64%) of the differential metabolites of the milk and serum from H1–H4 shared differential metabolites, with milk alone accounting for 47 (28.48%) and serum alone accounting for 112 (67.88%) of the differential metabolites (Figure 5A). A Spearman’s correlation analysis of these six shared differential metabolites revealed that hippuric acid in the serum was highly and significantly correlated with citric acid in the milk and significantly correlated with nicotinic acid in the milk (Figure 5B). Hippuric acid itself was significantly negatively correlated between the two body fluids. At the same time, the physiological indicators with significant differences in the H1–H4 serum or milk were screened based on the previous results, and Mantel’s correlation was performed with these six shared differential metabolites (Figure 5C). The physiological indicators that differed significantly between H1–H4 were lactose in the milk, total protein and lipase in the serum, and the three serum free amino acids histidine, glutamine, and glycine, respectively. Cholesterol in the serum was found to be significantly correlated with glycine, and also cholesterol in the milk was significantly correlated with histidine. Meanwhile, in the serum, there is a highly significant correlation between glutamine and pseudouridine, while in the milk, there is a significant correlation between glycine and citric acid. The analysis of the identification intensities of cholesterol and pseudouridine from the H1–H4 serum, and cholesterol and citric acid in the milk revealed that cholesterol showed opposite trends in the serum and milk, with cholesterol increased with parity in the serum but decreased with parity in the milk (Figure 5D). Pseudouridine in the serum and citric acid in the milk were both highest in H1. 

## 4. Discussion

The parity significantly affected the milkability and milk and health traits [21]. Consistent with many previous studies showing that the milk yield of primiparous cows is generally lower than that of multiparous cows [22], no significant differences in the milk yield among the four parturients were observed in this study, which may be due to different farm management conditions and the selection of the lactation period (within 100 days of early lactation, in the studies above). However, it is essential to note that we observed a significant change in lactose between the primiparous and multiparous cows, with H1 being the highest and starting to decrease with subsequent parity, which is consistent with previous findings [23,24]. Also, the serum lipase levels were significantly lower in H3 than in H1, probably due to more active lipid mobilization in the multiparous cows than in the primiparous cows [25], which is used to participate in increasing endogenous glucose production and hepatic glucose output for milk synthesis and to adapt to post-calving fuel oxidation [26]. It is worth noting that the serum GLU level had an increasing trend with the increase in parity but this was not significant; it is still possible to suggest that the glucose produced by catabolism in H1 could be used as a precursor to further participate in the energy metabolism of the milk and lactose synthesis process [27]. Meanwhile, the milk protein in the present study did not show a decreasing trend with increasing parity, as in the previous study [28]. However, the total protein in the serum showed a significant increasing trend with increasing parity, which is consistent with a previous study that suggested that a lower serum total protein concentration in dairy cows may be due to the availability of amino acids for milk production [29]. Thus, this suggests that cows with low parity may be more active in the metabolic process of milk protein synthesis using TP. 

Histidine is a limiting amino acid during lactation that affects milk production and milk protein synthesis in cows [30], and in the serum free amino acid results, the histidine content in H1 was significantly lower than in the later multiparity, which means that more attention should be paid to dietary protein supplementation or additional limiting amino acids in the feeding management to meet the metabolic requirements of primiparous dairy cows [31]. The plasma glutamate concentration was previously found to decrease in early lactation [32] and during feeding restriction experiments [33], and the Billa study demonstrated a strong correlation between the glutamate concentration and negative energy balance (NEB) and energy metabolism in dairy cows [34]. Combined with the lower glutamate level in H1 in this study, it is again suggested that more attention should be paid to the energy requirements of primiparous dairy cows to achieve a better milk production performance. Moreover, serum glycine has been previously reported to be positively correlated with milk production in early lactation [35]. The serum glycine–alanine (Gly/Ala) ratio is considered a potential marker to assess the nutritional status of periparturient cows [36]. In the present study, we examined the relationship between the glycine and alanine levels in the serum of the Holstein cows in the middle of lactation at different parities. Both the glycine and alanine levels in serum, as well as their ratio, were higher in H4. This suggests that cows experiencing multiple production events had a more stable physical performance in milk production and metabolic status. Our results are consistent with the study that showed that cholesterol is significantly higher in the serum of multiparous cows than in primiparous cows [37], and also that several of the amino acids mentioned above that were significantly different in the H1–H4 sera were all significantly correlated with cholesterol. Cholesterol is commonly used as an essential marker of blood metabolism and is positively correlated with milk fat levels [38], is strongly associated with a negative energy balance in dairy cows and is significantly different before and after calving [39,40,41]. Previously, cholesterol levels in heat-stressed cows were shown to increase with increasing doses of L-theanine supplementation in the diet [42], but studies focusing on cholesterol effects on amino acid metabolism have been relatively scarce in dairy cows. The results of this study provide a new insight that more attention can be paid to the effect of cholesterol on amino acid metabolism rather than just limiting it to the blood biochemical index itself, which more controlled experiments in the future should verify.

Compared to previous studies that only measured apparent parameters such as milk quality indicators and blood biochemical indicators [43,44,45], we also used metabolomics to systematically evaluate the milk and serum, providing a metabolic snapshot of Holstein cows at the same lactation period but different parities. Significant differences in lactose between parities were mentioned in the previous milk quality test results, and lactose as a disaccharide composed of glucose and galactose, which is the main carbohydrate in the milk of most placental mammals, accounting for more than 80% of all its carbohydrates [46]. Lactose is unique in that it is synthesized only by epithelial cells in the mammary gland [47], and the precursors delivered to the mammary epithelial cells to support lactose synthesis come primarily from glucose in the blood. Although the glucose concentrations measured directly in the serum in this study did not differ among the Holstein cows of the four parities, the milk quality results showed higher levels of lactose in H1. For this phenomenon, we found more sensitive metabolomic assays in which Alpha-D-Glucose had a higher intensity in the serum of H1, which could also be involved in the reaction as a lactose synthesis precursor [48]. In addition, lactose synthesis is a physiological process with a high energy requirement [49], while the glucose that makes up lactose is directly involved in glycolysis to supply the body with energy [50]. Previous published studies have mentioned that glucose in the blood is not the only precursor for lactose synthesis, but glycerol and galactose are alternative carbon sources for lactose synthesis [51]. The precursors for lactose synthesis are all derived from blood flowing through the mammary gland. In this study, a total of 12 differential metabolites were identified in the serum carbohydrate category, half of which were identified with a higher intensity in H1, including alpha-D-glucose, citric acid, D-glucuronic acid, fructose-1P, N-acetyl-D-glucosamine, and N-Glycolylneuraminic acid. Among them, alpha-D-glucose serves as the starting point of glycolysis, and fructose-1P is indirectly involved in the glycolytic reaction by generating glyceraldehyde and dihydroxyacetone phosphate (DHAP) in the presence of aldolase B hydrolyzes [52], and citric acid serves as the starting point of the tricarboxylic acid cycle, linking upstream glycolytic reactions for direct energy supply to the body [53]. In addition, we found that fructose 1,6-bisphosphate, which is involved in energy metabolism in serum, was also identified at a higher intensity in H1. Fructose 1,6-bisphosphate is an important intermediate in glycolysis and will be hydrolyzed to glyceraldehyde-3-phosphate in the presence of aldolase with DHAP to participate in subsequent energy metabolism reactions [54]. In contrast to previous studies that have focused more on changes in energy balance and glucose metabolism in periparturient cows, our results suggest that differences in glucose metabolism for Holstein cows in mid-lactation between parities 1 and 4 are also important, and the positive effects of metabolic physiology on the milk quality can be maximized by developing more rational dietary management formulas for cows in mid-lactation at different parities.

**Figure 6:**
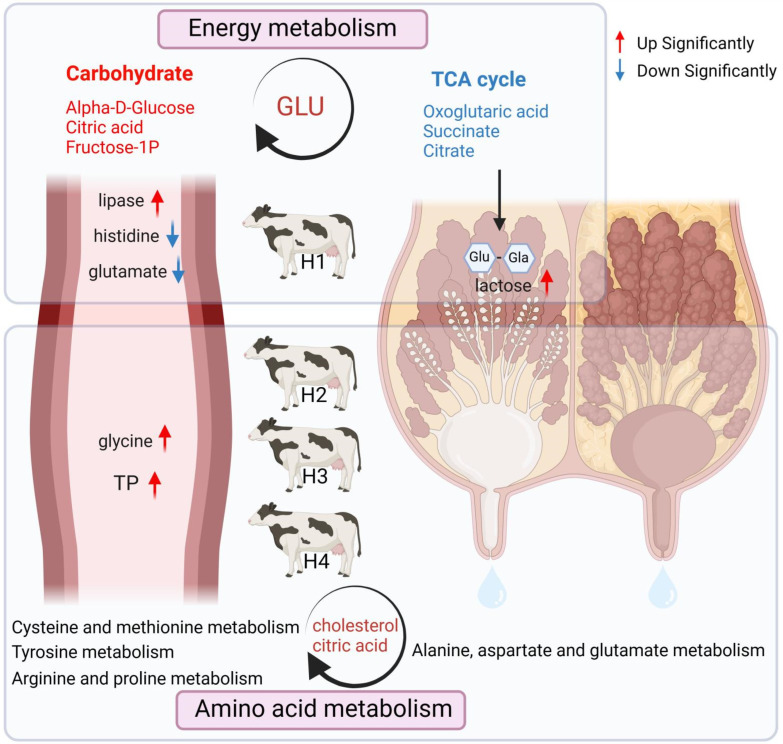
Summary diagram showing the main results from the present work. H1 exhibits a higher energy metabolism demand, as evidenced by the higher levels of carbohydrate metabolism in the H1-B, which can stimulate the TCA cycle in milk through the common intermediate product glucose, resulting in higher lactose content in H1-M. On the other hand, H2–H4 show higher levels of total protein and free amino acids in the serum, indicating a more active amino acid metabolism.

Some of the six differential impact pathways identified in the serum, including tyrosine metabolism, cysteine and methionine metabolism, and arginine and proline metabolism, were involved in amino acid metabolism, which was consistent with the identification of free amino acids in the serum, indicating that there are some fluctuations and differences in the amino acid content in the serum of Holstein cows between parities, but they do not affect the composition and proportion of amino acids in milk. Similarly, among the only two metabolic pathways identified as being different in the milk, the TCA cycle, which has a higher impact index, is an important pathway for energy metabolism in the animal [55], and the co-use of lactose synthesis precursor glucose for metabolism. Glyoxylate and dicarboxylate metabolism is closely related to the TCA cycle. Also, as a variant of energy metabolism, glyoxylate and dicarboxylic acid metabolism use intermediates such as isocitrate and α-ketoglutarate that participate in the TCA cycle to regulate amino acid metabolism [56]. Three metabolites, oxoglutaric acid, succinate, and citrate, which are critical intermediates of the TCA cycle, were also found to be at higher levels in H1-M. Although the TCA cycle occurs mainly in tissue cells and is difficult to find in milk, it can be found and enriched in the mammary gland [57]. Therefore, these metabolites have the potential to pass through the mammary gland into milk. Notably, a more active TCA cycle consumes more glucose [58], which is also used for lactose synthesis, as mentioned earlier, and more attention should be paid to balancing the glucose requirements of H1 during feeding management (Figure 6). However, further studies are needed to investigate the underlying mechanisms by which blood metabolites affect milk production and quality, and adjust nutritional strategies to achieve these outcomes. Furthermore, integrating metabolomics data with other omics data, such as genomics, transcriptomics, and proteomics, is essential to gain a more holistic understanding of the metabolic changes that occur with increasing parity in dairy cows. Future research efforts should focus on elucidating the underlying mechanisms and determining optimal nutritional strategies to further optimize milk production and quality in this economically important species.

## 5. Conclusions

By analyzing the changes in the milk and serum of Holstein cows from parities 1 to 4, we found that the milk from H1 had the highest lactose content, which related to more active carbohydrate metabolism in the serum of H1. The composition of the milk hydrolyzed amino acids did not differ significantly among different parities. The total protein and free amino acids with significant differences in the serum of H1–H4 exhibited a consistent pattern across the different parities. The 53 and 118 differential metabolites identified in the milk and serum from H1–H4 could be classified into seven and nine categories based on the KEGG database, respectively. Among the six shared differential metabolites identified by the serum and milk metabolomics, cholesterol and citric acid were found to regulate multiple metabolic pathways, which were closely related to amino acid metabolism. These results reveal the relationship between the serum metabolome of Holstein cows and the production of milk. The findings indicate differences in the lactation energy of Holstein cows at different parities. Blood metabolites play an important role in regulating the milk yield and milk quality of Holstein cows. This further highlights the importance of addressing nutritional needs at these stages to improve lactation performance.

## Figures and Tables

**Table 1:** Milk quality of Holstein dairy cattle in different parities.

Item ^1^	Group ^2^				SEM ^3^	*p*-Value ^4^		
	H1	H2	H3	H4		T	L	Q
BW (kg)	567	576	571	566	3.49	0.782	0.755	0.334
DMI (kg/d)	22.62	23.14	23.76	23.19	0.21	0.248	0.052	0.927
Milk yield (kg/d)	31.63	27.91	33.01	29.79	1.21	0.533	0.968	0.921
Somatic cell count (×1000 cells/mL)	24.5	42.25	29.43	35.83	4.36	0.574	0.589	0.551
Milk fat (%)	4.15	4.26	4.24	4.25	0.19	0.997	0.888	0.906
Milk protein (%)	4.13	4.17	4.00	4.11	0.07	0.887	0.741	0.806
Lactose (%)	5.19 ^a^	4.97 ^b^	4.94 ^b^	5.09 ^ab^	0.04	0.036	0.253	0.009
Dry matter without fat (%)	9.50	9.30	9.11	9.36	0.07	0.221	0.301	0.107
Urea Nitrogen (%)	11.59	12.63	12.44	11.86	0.17	0.117	0.663	0.021

^1^ Abbreviations: BW = body weight; DMI= dry matter intake; ^2^ H1, first-parity Holstein dairy cattle; H2, second-parity Holstein dairy cattle; H3, third-parity Holstein dairy cattle; H4, fourth-parity Holstein dairy cattle. ^a,b^ Means within a row with different superscripts differ (*p* < 0.05). ^3^ SEM was standard error of means. ^4^ T = treat; L = linear; Q = quadratic.

**Table 2:** Serum biochemical parameters of Holstein dairy cattle in different parities.

Item	Group ^1^				SEM ^2^	*p*-Value ^3^		
	H1	H2	H3	H4		T	L	Q
Glucose (mM)	3.41	3.56	3.69	3.91	0.07	0.081	0.012	0.807
Total protein (g/L)	82.60 ^b^	82.06 ^b^	87.00 ^ab^	91.78 ^a^	1.28	0.013	0.002	0.252
Albumin (g/L)	41.22	39.5	42.39	41.81	0.64	0.481	0.408	0.663
Blood urea nitrogen (mM)	5.73	5.89	6.14	5.96	0.19	0.909	0.585	0.677
Triglyceride (mM)	0.13	0.15	0.13	0.12	0.01	0.172	0.206	0.099
Low-density lipoprotein (mM)	2.77	2.65	2.61	2.59	0.13	0.965	0.634	0.853
High-density lipoprotein (mM)	3.95	3.77	3.95	3.79	0.10	0.884	0.749	0.963
Lipase (U/L)	17.11 ^b^	20.20 ^a^	20.03 ^a^	19.93 ^a^	0.41	0.022	0.291	0.083
Serum ammonia (μM)	270.68	259.39	272.00	265.82	5.28	0.859	0.967	0.817

^1^ H1, first-parity Holstein dairy cattle; H2, second-parity Holstein dairy cattle; H3, third-parity Holstein dairy cattle; H4, fourth-parity Holstein dairy cattle. ^a,b^ Means within a row with different superscripts differ (*p* < 0.05). ^2^ SEM was standard error of means. ^3^ T = treat; L = linear; Q = quadratic.

**Table 3:** Free amino acid content (μg/mL) in serum from Holstein cows of different parities.

Item ^1^	Group ^2^				SEM ^3^	*p*-Value ^4^		
	H1	H2	H3	H4		T	L	Q
EAAs	115.64	126.96	126.50	120.44	2.99	0.495	0.598	0.164
Threonine	25.81	25.89	25.06	24.36	0.48	0.658	0.230	0.692
Valine	26.80	29.22	29.88	28.72	0.91	0.650	0.431	0.346
Methionine	3.83	3.28	3.45	3.89	0.20	0.702	0.857	0.246
Isoleucine	12.87	14.19	13.25	12.72	0.43	0.681	0.713	0.300
Leucine	16.33	18.69	19.01	17.18	0.65	0.413	0.613	0.120
Phenylalanine	7.52	7.32	7.81	6.98	0.19	0.522	0.528	0.449
Lysine	14.22	18.54	16.88	16.49	0.57	0.055	0.267	0.034
Histidine	8.26 ^c^	9.81 ^b^	11.16 ^a^	10.09 ^b^	0.23	<0.001	<0.001	<0.001
NEAAs	133.92	130.61	143.88	142.70	2.88	0.310	0.119	0.854
Aspartate	2.19	2.12	2.20	2.19	0.08	0.989	0.913	0.853
Serine	9.53	9.63	10.81	11.24	0.29	0.075	0.011	0.767
Glutamate	24.76 ^b^	24.74 ^ab^	30.56 ^a^	25.85 ^ab^	0.86	0.039	0.193	0.149
Glycine	26.09 ^ab^	21.65 ^b^	27.33 ^ab^	29.65 ^a^	0.95	0.031	0.036	0.060
Alanine	23.68	23.28	22.87	23.99	0.52	0.885	0.912	0.484
Cysteine	3.06	2.73	2.66	2.90	0.12	0.641	0.606	0.257
Tyrosine	7.69	8.26	7.66	7.43	0.31	0.844	0.619	0.532
Arginine	25.63	25.29	28.73	29.36	0.85	0.220	0.051	0.774
Proline	11.29	12.90	11.06	10.08	0.57	0.438	0.277	0.269
TAAs	249.56	257.57	270.38	263.14	5.30	0.545	0.259	0.487
EAA/TAA	0.46	0.49	0.47	0.46	0.00	0.065	0.336	0.027

^1^ EAAs, essential amino acids; NEAAs, non-essential amino acids; TAA, total amino acid; EAA/TAA, the ratio of essential amino acids to total amino acids. ^2^ H1, first-parity Holstein dairy cattle; H2, second-parity Holstein dairy cattle; H3, third-parity Holstein dairy cattle; H4, fourth-parity Holstein dairy cattle. ^a–c^ Means within a row with different superscripts differ (*p* < 0.05). ^3^ SEM was standard error of means. ^4^ T = treat; L = linear; Q = quadratic.

## Data Availability

The authors confirm that the data supporting the findings of this study are available within the article.

## Supplementary Materials

The following supporting information can be downloaded at https://www.mdpi.com/article/10.3390/ani14030407/s1, Table S1: Ingredients and chemical composition of experimental diets; Table S2: Hydrolyzed amino acid content (g/mL) in milk from Holstein cows of different parities; Table S3: Multivariate statistical analysis parameters from untargeted metabolomic of 1–4 parity Holstein cows; Table S4: Metabolites identified from 1–4 parity Holstein cows milk by UPLC-MS/MS analysis technology; Table S5: Metabolites identified from 1–4 parity Holstein cows serum by UPLC-MS/MS analysis technology.

## Abbreviations

ADF	Acid detergent fiber
AOAC	Official Analytical Chemists methodologies
ALB	Albumin
BUN	Blood urea nitrogen Burea nitrogen
Ca	Calcium
CP	Crude protein
DIM	Days in milk
DM	Dry matter
EAA	Essential amino acid
GE	Gross energy
GLU	Glucose
HCA	Hierarchical cluster analysis
HCL	Hydrochloric acid
HDL-C	High-density lipoprotein cholesterol
KEGG	Genes and Genomes pathway database
LDL-C	Low-density lipoprotein cholesterol
LIP	Lipase
NDF	Neutral detergent fiber
NEAA	Non-essential amino acids
NH3L	Blood ammonia
OPLS-DA	Orthogonal partial least squares discriminate analysis
P	Phosphorus
RT	Retention time
SCC	Somatic cell count
SEM	Standard error of means
TAA	Total amino acid
TG	Triglyceride
TMR	Total mixed rations
TP	Total protein
UPLC-MS/MS	Ultra-high-resolution high-performance liquid chromatography with tandem mass spectrometry
VIP	Variable importance in the projection
