# Supplementary material for: Metabolic Changes in Serum and Milk of Holstein Cows in Their First to Fourth Parity Revealed by Biochemical Analysis and Untargeted Metabolomics
Source: Animals (Basel). 2024 Jan 26;14(3):407. doi: 10.3390/ani14030407 (PMC10854930; doi:10.3390/ani14030407)
Supplement: Supplementary file 1 [file animals-14-00407-s001.zip › animals-2789495-supplementary.pdf]

Metabolic changes in serum and milk of Holstein cows in their first to fourth parity revealed by biochemical analysis and untargeted metabolomics

Zixin Liu [1,2†], Aoyu Jiang [1,2†], Xiaokang Lv [1,2,3], Qingqing Chen [1], Chuanshe Zhou [1,2*], Zhiliang Tan [1,2]

[1] CAS Key Laboratory for Agri-Ecological Processes in Subtropical Region, National Engineering Laboratory for Pollution CON and Waste Utilization in Livestock and Poultry Production, Hunan Provincial Key Laboratory of Animal Nutrition Physiology and Metabolic Process, Institute of Subtropical Agriculture, Chinese Academy of Sciences, Changsha 410125, China

[2] University of the Chinese Academy of Sciences, Beijing 100049, P. R. China

[3] College of Animal Science, Anhui Science and Technology University, Bengbu, 233100, P. R. China

† These authors contributed equally to this work.

* Correspondence: zcs@isa.ac.cn; Tel.: +86-731-84619795; Fax: +86-731-84612685

Table S1. Ingredients and chemical composition of experimental diets

Item	Content
Ingredients, % of diet DM	
Soybean meal	13
Cottonseed meal	2
Barley	5
Distillers Dried Grains with Solubles	2
Sprouting corn bran	2
Corn	21
Flaked maize	5
Pelleted beet pulp	2
Cottonseed	5.5
Oat hay	2.5
Alfalfa hay	11
Corn silage	23
Alfalfa silage	4.5
NaHCO3	0.5
Premix[1]	1
Total	100
Nutrient levels[2]	
DM	48.85
CP	15.14
NDF	35.22
ADF	37.57
EE	6.83
Ash	10.23
GE (MJ/kg)	16.56
Ca	0.78
P	0.36

[1] The premix provided the following per kg of diets：50 g Mg, 2.5 g Fe, 0.4 g Cu, 2 g Mn, 1.5 g Zn, 10 mg Se, 25 mg I, 5 mg Co, 500,000 IU vitamin A, 25,000 IU vitamin D, and 2500 IU vitamin E.

[2] DM = dry matter; CP = crude protein; NDF = neutral detergent fiber; ADF = acid detergent fiber; EE= ether extract; GE = gross energy; Ca = calcium; P = phosphorus.

Table S2. Hydrolyzed amino acid content (g/mL) in milk from Holstein cows of different parities

Item[1]	Group[2]				SEM[3]	P[4]		
	H1	H2	H3	H4		T	L	Q
EAA	1.48	1.53	1.54	1.53	0.041	0.953	0.642	0.766
Arginine	0.10	0.10	0.10	0.10	0.003	0.885	0.480	0.785
Threonine	0.12	0.13	0.13	0.13	0.003	0.701	0.366	0.525
Valine	0.20	0.21	0.21	0.21	0.006	0.860	0.524	0.613
Methionine	0.08	0.07	0.08	0.07	0.002	0.991	0.956	0.932
Isoleucine	0.18	0.19	0.19	0.19	0.005	0.990	0.924	0.774
Leucine	0.31	0.31	0.32	0.32	0.008	0.967	0.632	0.926
Phenylalanine	0.17	0.17	0.17	0.17	0.005	0.999	0.922	0.923
Lysine	0.24	0.26	0.25	0.25	0.008	0.801	0.433	0.594
Histidine	0.09	0.09	0.10	0.09	0.002	0.988	0.852	0.808
NEAA	1.45	1.53	1.55	1.63	0.045	0.505	0.135	0.945
Aspartate	0.21	0.22	0.22	0.23	0.005	0.761	0.294	0.911
Serine	0.11	0.13	0.13	0.14	0.004	0.239	0.087	0.344
Glutamate	0.47	0.51	0.52	0.53	0.017	0.532	0.158	0.783
Glycine	0.06	0.06	0.06	0.06	0.001	0.841	0.408	0.796
Alanine	0.10	0.10	0.11	0.11	0.002	0.995	0.822	0.970
Tyrosine	0.20	0.21	0.21	0.21	0.006	0.860	0.524	0.613
Proline	0.30	0.30	0.31	0.36	0.015	0.353	0.125	0.332
TAA	2.83	2.95	2.98	3.05	0.080	0.778	0.312	0.919
EAA/TAA	0.52	0.52	0.52	0.50	0.003	0.131	0.026	0.365

[1] EAA, essential amino acids; NEAA, non-essential amino acids; TAA, total amino acid; EAA/TAA, the ratio of essential amino acids to total amino acids.

[2] H1, first parity Holstein dairy cattle; H2, second parity Holstein dairy cattle; H3, third parity Holstein dairy cattle; H4, fourth parity Holstein dairy cattle.

[3] SEM was standard error of means.

[4] T = Treat; L = linear; Q = quadratic.

Table S3. Multivariate statistical analysis parameters from untargeted metabolomic of 1-4 parity Holstein cows

Item	Mode	Sample pair[1]	Statistical model[2]	R2X(cumulative)[3]	R2Y(cumulative)[4]	Q2(cumulative)[5]
Milk	Positive ion mode	S1 vs S2 vs S3 vs S4	OPLS-DA	0.707	0.833	0.292
	Negative ion mode	S1 vs S2 vs S3 vs S4	OPLS-DA	0.322	0.953	0.658
Serum	Positive ion mode	S1 vs S2 vs S3 vs S4	OPLS-DA	0.632	0.995	0.897
	Negative ion mode	S1 vs S2 vs S3 vs S4	OPLS-DA	0.364	0.987	0.763

[1] S1, first parity Holstein cows; S2, second parity Holstein cows; S3, third parity Holstein cows; S4, fourth parity Holstein cows.

[2] OPLS-DA, orthogonal partial least square discriminant analysis.

[3] R2X means the rate of interpretation of the X matrix by the model.

[4] R2Y means the rate of interpretation of the Y matrix by the model.

[5] Q2 represents the predictive ability of the model.

Table S4. Metabolites identified from 1-4 parity Holstein cows milk by UPLC-MS/MS analysis technology

Num	Metabolite[1]	m/z[2]	RT(s)[3]	Formula[4]	KEGG[5]	Type[6]
1	Metoclopramide	300.14	602.25	C14H22ClN3O2	C07868	+
2	Diaminopimelic acid	173.09	550.93	C7H14N2O4	C00666	+
3	Thyrotropin releasing hormone	362.32	724.25	C16H22N6O4	C03958	+
4	3-Indoleacetonitrile	156.12	778.21	C10H8N2	C02938	+
5	Acetaminophen	151.06	210.76	C8H9NO2	C06804	+
6	Adenosine	268.10	239.49	C10H13N5O4	C00212	+
7	17a-Hydroxypregnenolone	333.24	835.53	C21H32O3	C05138	+
8	Mirtazapine	266.17	688.13	C17H19N3	C07570	+
9	Isovaleric acid	102.09	397.06	C5H10O2	C08262	+
10	Racemethionine	150.06	121.41	C5H11NO2S	C01733	+
11	8-Amino-7-oxononanoate	170.12	483.30	C9H17NO3	C01092	+
12	Succinic acid semialdehyde	103.04	86.26	C4H6O3	C00232	+
13	3-Methyloxindole	148.07	402.58	C9H9NO	C02366	+
14	Methoxamine	194.12	515.02	C11H17NO3	C07513	+
15	N-Acetyl-D-glucosamine	204.09	93.90	C8H15NO6	C00140	+
16	Cholesterol	369.35	796.44	C27H46O	C00187	+
17	5-Hydroxymethyluracil	142.03	67.79	C5H6N2O3	C03088	+
18	Neocembrene	273.25	742.33	C20H32	C09140	+
19	Cortexolone	346.33	787.79	C21H30O4	C05488	+
20	Hippuric acid	178.05	333.72	C9H9NO3	C01586	-
21	4-Nitrophenol	138.02	616.23	C6H5NO3	C00870	-
22	Citramalic acid	147.03	72.66	C5H8O5	C00815	-
23	Salicyluric acid	194.05	380.09	C9H9NO4	C07588	-
24	Spermidine	143.92	57.46	C7H19N3	C00315	-
25	3-(2-Hydroxyphenyl)propanoic acid	146.97	299.46	C9H10O3	C01198	-
26	Oxoglutaric acid	145.01	73.36	C5H6O5	C00026	-
27	Maleic acid	114.99	98.76	C4H4O4	C01384	-
28	Phenylacetylglycine	192.07	371.18	C10H11NO3	C05598	-
29	Cellobiose	341.11	86.58	C12H22O11	C00185	-
30	L-Gulose	179.06	435.19	C6H12O6	C15923	-
31	trans-Aconitic acid	155.00	69.11	C6H6O6	C02341	-
32	N-Acetylaspartylglutamic acid	303.08	151.68	C11H16N2O8	C12270	-
33	Citric acid	191.02	69.39	C6H8O7	C00158	-
34	D-Mannose	161.04	232.23	C6H12O6	C00159	-
35	dCMP	306.06	139.26	C9H14N3O7P	C00239	-
36	Mesaconate	129.02	72.53	C5H6O4	C01732	-
37	Glutaric acid	131.00	69.39	C5H8O4	C00489	-
38	13S-hydroxyoctadecadienoic acid	295.23	835.65	C18H32O3	C14762	-
39	Glycerylphosphorylethanolamine	214.05	82.48	C5H14NO6P	C01233	-
40	2-Furoate	111.01	69.40	C5H4O3	C01546	-
41	D-Galactaro-1,5-lactone	191.02	96.10	C6H8O7	C20889	-
42	Uric acid	167.02	94.01	C5H4N4O3	C00366	-

43	L-4-Hydroxyphenylglycine	166.05	194.58	C8H9NO3	C12323	-
44	Glycerol 3-phosphate	153.00	82.22	C3H9O6P	C00093	-
45	Gentisic acid	153.02	437.24	C7H6O4	C00628	-
46	Pseudouridine	243.06	139.05	C9H12N2O6	C02067	-
47	Cyclic AMP	328.04	317.18	C10H12N5O6P	C00575	-
48	5-Methoxyindoleacetate	204.07	446.17	C11H11NO3	C05660	-
49	Acetylcholine chloride	180.97	81.82	C7H16NO2. Cl	C08201	-
50	2-Pyrocatechuic acid	154.02	437.24	C7H6O4	C00196	-
51	Succinic acid	117.02	73.63	C4H6O4	C00042	-
52	Nicotinuric acid	180.06	333.67	C8H8N2O3	C05380	-
53	2-(Methylamino)benzoic acid	150.06	380.01	C8H9NO2	C03005	-
1	Metoclopramide	300.14	602.25	C14H22ClN3O2	C07868	+
2	Diaminopimelic acid	173.09	550.93	C7H14N2O4	C00666	+
3	Thyrotropin releasing hormone	362.32	724.25	C16H22N6O4	C03958	+
4	3-Indoleacetonitrile	156.12	778.21	C10H8N2	C02938	+
5	Acetaminophen	151.06	210.76	C8H9NO2	C06804	+
6	Adenosine	268.10	239.49	C10H13N5O4	C00212	+
7	17a-Hydroxypregnenolone	333.24	835.53	C21H32O3	C05138	+
8	Mirtazapine	266.17	688.13	C17H19N3	C07570	+
9	Isovaleric acid	102.09	397.06	C5H10O2	C08262	+
10	Racemethionine	150.06	121.41	C5H11NO2S	C01733	+
11	8-Amino-7-oxononanoate	170.12	483.30	C9H17NO3	C01092	+
12	Succinic acid semialdehyde	103.04	86.26	C4H6O3	C00232	+
13	3-Methyloxindole	148.07	402.58	C9H9NO	C02366	+
14	Methoxamine	194.12	515.02	C11H17NO3	C07513	+
15	N-Acetyl-D-glucosamine	204.09	93.90	C8H15NO6	C00140	+
16	Cholesterol	369.35	796.44	C27H46O	C00187	+
17	5-Hydroxymethyluracil	142.03	67.79	C5H6N2O3	C03088	+
18	Neocembrene	273.25	742.33	C20H32	C09140	+
19	Cortexolone	346.33	787.79	C21H30O4	C05488	+
20	Hippuric acid	178.05	333.72	C9H9NO3	C01586	-
21	4-Nitrophenol	138.02	616.23	C6H5NO3	C00870	-
22	Citramalic acid	147.03	72.66	C5H8O5	C00815	-
23	Salicyluric acid	194.05	380.09	C9H9NO4	C07588	-
24	Spermidine	143.92	57.46	C7H19N3	C00315	-
25	3-(2-Hydroxyphenyl)propanoic acid	146.97	299.46	C9H10O3	C01198	-
26	Oxoglutaric acid	145.01	73.36	C5H6O5	C00026	-
27	Maleic acid	114.99	98.76	C4H4O4	C01384	-
28	Phenylacetylglycine	192.07	371.18	C10H11NO3	C05598	-
29	Cellobiose	341.11	86.58	C12H22O11	C00185	-
30	L-Gulose	179.06	435.19	C6H12O6	C15923	-
31	trans-Aconitic acid	155.00	69.11	C6H6O6	C02341	-
32	N-Acetylaspartylglutamic acid	303.08	151.68	C11H16N2O8	C12270	-
33	Citric acid	191.02	69.39	C6H8O7	C00158	-

34	D-Mannose	161.04	232.23	C6H12O6	C00159	-
35	dCMP	306.06	139.26	C9H14N3O7P	C00239	-
36	Mesaconate	129.02	72.53	C5H6O4	C01732	-
37	Glutaric acid	131.00	69.39	C5H8O4	C00489	-
38	13S-hydroxyoctadecadienoic acid	295.23	835.65	C18H32O3	C14762	-
39	Glycerylphosphorylethanolamine	214.05	82.48	C5H14NO6P	C01233	-
40	2-Furoate	111.01	69.40	C5H4O3	C01546	-
41	D-Galactaro-1,5-lactone	191.02	96.10	C6H8O7	C20889	-
42	Uric acid	167.02	94.01	C5H4N4O3	C00366	-
43	L-4-Hydroxyphenylglycine	166.05	194.58	C8H9NO3	C12323	-
44	Glycerol 3-phosphate	153.00	82.22	C3H9O6P	C00093	-
45	Gentisic acid	153.02	437.24	C7H6O4	C00628	-
46	Pseudouridine	243.06	139.05	C9H12N2O6	C02067	-
47	Cyclic AMP	328.04	317.18	C10H12N5O6P	C00575	-
48	5-Methoxyindoleacetate	204.07	446.17	C11H11NO3	C05660	-
49	Acetylcholine chloride	180.97	81.82	C7H16NO2. Cl	C08201	-
50	2-Pyrocatechuic acid	154.02	437.24	C7H6O4	C00196	-
51	Succinic acid	117.02	73.63	C4H6O4	C00042	-
52	Nicotinuric acid	180.06	333.67	C8H8N2O3	C05380	-
53	2-(Methylamino)benzoic acid	150.06	380.01	C8H9NO2	C03005	-

[1] The name of the metabolite that matches in the secondary mass spectrum.

[2] Mass-to-charge ratio.

[3] Retention time.

[4] Molecular formulas of the metabolite.

[5]The identification number of the metabolite in the Kyoto Encyclopedia of Genes and Genomes (KEGG) database.

[6] Metabolite identified in positive mode is denoted as "+" and metabolite identified in negative mode is denoted as "-".

Table S5. Metabolites identified from 1-4 parity Holstein cows serum by UPLC-MS/MS analysis technology

Num	Metabolite[1]	m/z[2]	RT(s)[3]	Formula[4]	KEGG[5]	Type[6]
1	Catechol	111.02	838.43	C6H6O2	C00090	+
2	5,6-Dihydro-5-fluorouracil	133.03	140.17	C4H5FN2O2	C16630	+
3	4-Hydroxyphenylacetaldehyde	136.05	139.66	C8H8O2	C03765	+
4	Methyl (indol-3-yl)acetate	189.08	655.14	C11H11NO2	C20635	+
5	Benzamide	122.06	466.91	C7H7NO	C09815	+
6	Indican	294.94	78.70	C14H17NO6	C08481	+
7	Picolinic acid	124.09	756.94	C6H5NO2	C10164	+
8	N-Acetylputrescine	131.12	514.32	C6H14N2O	C02714	+
9	O-Phosphoethanolamine	141.96	67.41	C2H8NO4P	C00346	+
10	cis-4-Hydroxy-D-proline	132.07	86.37	C5H9NO3	C03440	+
11	GMP	362.92	78.61	C10H14N5O8P	C00144	+
12	Uracil	113.03	173.52	C4H4N2O2	C00106	+
13	7-Methylguanine	166.07	146.08	C6H7N5O	C02242	+
14	Sphingosine	300.29	770.26	C18H37NO2	C00319	+
15	Nicotinic acid	124.04	137.08	C6H5NO2	C00253	+
16	N5-Methyl-L-glutamine	161.09	96.80	C6H12N2O3	C03153	+
17	Qing Hau Sau	282.14	835.18	C15H22O5	C09538	+
18	Caffeine	195.08	760.96	C8H10N4O2	C07481	+
19	Norepinephrine	169.98	742.69	C8H11NO3	C00547	+
20	Phosphoglycolic acid	156.99	785.49	C2H5O6P	C00988	+
21	L-Fucose	164.07	262.40	C6H12O5	C00507	+
22	Theobromine	181.07	463.99	C7H8N4O2	C07480	+
23	Indolelactic acid	206.08	550.16	C11H11NO3	C02043	+
24	Benzoyl phosphate	184.99	231.11	C7H7O5P	C06206	+
25	3-Sulfinylpyruvic acid	134.97	69.67	C3H4O5S	C05527	+
26	N-Acetylhistidine	198.09	99.77	C8H11N3O3	C02997	+
27	Biotin	227.08	396.41	C10H16N2O3S	C00120	+
28	Aspirin	163.04	709.44	C9H8O4	C01405	+
29	Metoclopramide	300.14	609.37	C14H22ClN3O2	C07868	+
30	Hydroxypyruvic acid	105.04	464.28	C3H4O4	C00168	+
31	Trifolirhizin	447.13	707.10	C22H22O10	C10538	+
32	L-Proline	116.07	95.41	C5H9NO2	C00148	+
33	Acetylcysteine	163.00	62.56	C5H9NO3S	C06809	+
34	Imidazolepropionic acid	141.07	99.76	C6H8N2O2	C20522	+
35	4-Oxoglutaramate	145.05	63.83	C5H7NO4	C05572	+
36	(2R,3R)-3-Methylglutamyl-5-semialdehyde-N6-lysine	274.19	95.77	C12H23N3O4	C20279	+
37	Betonicine	142.09	360.96	C7H13NO3	C08269	+
38	L-Methionine	150.06	140.23	C5H11NO2S	C00073	+
39	Butyryl-L-carnitine	232.15	368.83	C11H21NO4	C02862	+
40	Dodecanoic acid	200.16	649.22	C12H24O2	C02679	+

41	Porphobilinogen	226.18	691.18	C10H14N2O4	C00931	+
42	Nicotinuric acid	181.06	421.97	C8H8N2O3	C05380	+
43	2-Oxo-4-methylthiobutanoic acid	149.02	457.85	C5H8O3S	C01180	+
44	Epigallocatechin gallate	459.09	458.26	C22H18O11	C09731	+
45	Rimantadine	180.17	691.14	C12H21N	C07236	+
46	Vanillylmandelic acid	198.19	691.22	C9H10O5	C05584	+
47	Isophorone	139.11	712.68	C9H14O	C14743	+
48	Eugenol	165.09	735.82	C10H12O2	C10453	+
49	L-Carnitine	162.11	88.39	C7H15NO3	C00318	+
50	Mirtazapine	266.17	691.23	C17H19N3	C07570	+
51	Lithocholic acid	359.29	760.79	C24H40O3	C03990	+
52	Sorbitol	182.98	40.37	C6H14O6	C00794	+
53	12-Hydroxydodecanoic acid	216.20	657.28	C12H24O3	C08317	+
54	Sphingosine 1-phosphate	380.26	795.88	C18H38NO5P	C06124	+
55	26-Hydroxyecdysone	480.27	667.15	C27H44O7	C16499	+
56	L-Prolinamide	115.09	73.58	C5H10N2O	C19781	+
57	Cholesterol	369.36	810.39	C27H46O	C00187	+
58	Hippuric acid	162.05	463.76	C9H9NO3	C01586	+
59	Dihydrouracil	115.05	136.32	C4H6N2O2	C00429	+
60	Ipratropium	331.21	598.82	C20H30NO3	C07052	+
61	L-Valine	118.09	104.33	C5H11NO2	C00183	+
62	Caffeic acid	163.15	560.17	C9H8O4	C01197	+
63	HC-toxin	437.23	458.66	C21H32N4O6	C15676	+
64	Allocholic acid	391.28	761.90	C24H40O5	C00695	+
65	Myclobutanil	289.13	431.05	C15H17ClN4	C18477	+
66	Dehydroepiandrosterone	289.16	460.58	C19H28O2	C01227	+
67	1-palmitoylglycerophosphocho line	496.33	759.97	C24H51NO7P	C04102	+
68	Se-Methylselenocysteine	182.98	148.93	C4H9NO2Se	C05689	+
69	L-Glutamic acid	148.06	87.74	C5H9NO4	C00025	+
70	N-Glycolylneuraminic acid	324.09	78.70	C11H19NO10	C03410	-
71	3-Dehydroshikimate	171.03	88.46	C7H8O5	C02637	-
72	N3-Fumaroyl-L-2,3-diaminopr opanoate	201.06	88.11	C7H10N2O5	C20961	-
73	Beta-Guanidinopropionic acid	131.07	253.90	C4H9N3O2	C03065	-
74	Alpha-D-Glucose	161.04	636.41	C6H12O6	C00267	-
75	cis-3-(Carboxy-ethyl)-3,5-cycl o-hexadiene-1,2-diol	165.06	355.36	C9H12O4	C11588	-
76	Fructose-1P	161.04	708.70	C6H12O6	C10906	-
77	Leukotriene F4	549.28	608.77	C28H44N2O8S	C06462	-
78	17a-Estradiol	271.23	824.39	C18H24O2	C02537	-
79	Glyceric acid	105.02	79.30	C3H6O4	C00258	-
80	Hydroquinone	109.03	366.09	C6H6O2	C00530	-

81	Citric acid	191.02	70.22	C6H8O7	C00158	-
82	12,13-DHOME	295.23	824.66	C18H34O4	C14829	-
83	4-Hydroxyphenyl-2-propionic acid	165.06	417.91	C9H10O3	C03080	-
84	Dibutyl phthalate	277.14	715.27	C16H22O4	C14214	-
85	Adipic acid	145.05	577.45	C6H10O4	C06104	-
86	Homo-L-arginine	187.12	94.02	C7H16N4O2	C01924	-
87	L-Dopa	178.05	350.81	C9H11NO4	C00355	-
88	S-[(E)-N-Hydroxy(indol-3-yl)acetimidoyl]-L-glutathione	479.13	96.13	C20H25N5O7S	C21620	-
89	(2S)-2-[49]pentanoate	188.09	97.47	C8H15NO4	C06326	-
90	Guanidinosuccinic acid	174.96	56.24	C5H9N3O4	C03139	-
91	Vanylglycol	165.06	526.88	C9H12O4	C05594	-
92	beta-D-Glucosamine	160.06	94.76	C6H13NO5	C08349	-
93	Norspermidine	220.15	703.89	C6H17N3	C03375	-
94	Fructose 1,6-bisphosphate	339.20	825.17	C6H14O12P2	C00354	-
95	Deoxycorticosterone acetate	371.06	814.89	C23H32O4	C14554	-
96	Betaine	116.93	837.37	C5H11NO2	C00719	-
97	3-Hydroxyphenylacetic acid	151.04	364.47	C8H8O3	C05593	-
98	3-Methylthiopropionic acid	119.05	146.39	C4H8O2S	C08276	-
99	Pantetheine	278.12	135.29	C11H22N2O4S	C00831	-
100	Indoleacetic acid	174.06	700.20	C10H9NO2	C00954	-
101	Pyroglutamic acid	128.03	88.11	C5H7NO3	C01879	-
102	(13E)-11a-Hydroxy-9,15-dioxoprost-13-enoic acid	333.21	751.84	C20H32O5	C04654	-
103	Suberic acid	173.08	94.61	C8H14O4	C08278	-
104	p-Coumaroyl-D-glucose	326.10	368.52	C15H18O8	C16827	-
105	Galactitol	181.07	86.07	C6H14O6	C01697	-
106	Gingerol	293.18	809.53	C17H26O4	C10462	-
107	Azelaic acid	188.09	299.79	C9H16O4	C08261	-
108	9,10-Epoxyoctadecenoic acid	295.23	835.77	C18H32O3	C14825	-
109	(7Z,10Z,13Z,16Z,19Z)-Docosapentaenoic acid	329.25	820.14	C22H34O2	C16513	-
110	9(S)-HPODE	311.22	785.80	C18H32O4	C14827	-
111	2,4-Dinitrophenol	183.00	469.57	C6H4N2O5	C02496	-
112	N-Acetyl-D-glucosamine	221.15	703.98	C8H15NO6	C00140	-
113	D-Glucuronic acid	193.03	371.18	C6H10O7	C00191	-
114	Glycylleucine	169.10	326.07	C8H16N2O3	C02155	-
115	LysoPA(16:0/0:0)	391.22	833.90	C19H39O7P	C04036	-
116	Pseudouridine	243.06	152.93	C9H12N2O6	C02067	-
117	Dopamine	134.06	335.77	C8H11NO2	C03758	-
118	Folic acid	441.25	816.48	C19H19N7O6	C00504	-

Table S5. Metabolites identified from 1-4 parity Holstein cows serum by UPLC-MS/MS analysis technology

[1] The name of the metabolite that matches in the secondary mass spectrum.

[2] Mass-to-charge ratio.

[3] Retention time.

[4] Molecular formulas of the metabolite.

[5]The identification number of the metabolite in the Kyoto Encyclopedia of Genes and Genomes (KEGG) database.

[6] Metabolite identified in positive mode is denoted as "+" and metabolite identified in negative mode is denoted as "-".
